# Perceived posttraumatic growth after interpersonal trauma and subsequent well-being among young Colombian adults: A longitudinal analysis

**DOI:** 10.3389/fpsyg.2022.993609

**Published:** 2022-11-02

**Authors:** Zhuo Job Chen, Andrea Ortega Bechara, Richard G. Cowden, Everett L. Worthington

**Affiliations:** ^1^School of Nursing, University of North Carolina at Charlotte, Charlotte, NC, United States; ^2^Department of Psychology, Universidad del Sinú, Montería, Córdoba, Colombia; ^3^Human Flourishing Program, Harvard University, Cambridge, MA, United States; ^4^Department of Psychology, Virginia Commonwealth University, Richmond, VA, United States

**Keywords:** psychological trauma, posttraumatic growth, health, well-being, longitudinal studies, Colombia

## Abstract

Research has shown that people sometimes report self-perceived growth as a result of dealing with a potentially traumatic event, but relatively few methodologically rigorous studies have examined whether perceived posttraumatic growth is associated with improved subsequent well-being across a wide range of outcomes. In this three-wave longitudinal study of Colombian emerging adults (*n* = 636), we examined the associations of perceived posttraumatic growth with 17 well-being outcomes across domains of psychological well-being (i.e., self-rated mental health, meaning in life, sense of purpose, happiness, life satisfaction), psychological distress (i.e., anxiety symptoms, depression symptoms, subjective suffering), social well-being (i.e., content with relationships, satisfying relationships, loneliness), physical well-being (i.e., self-rated physical health, sleep quality), and character strengths (i.e., state hope, trait forgivingness, orientation to promote good, delayed gratification). Using an outcome-wide analytic design that adjusted for a range of covariates assessed in Wave 1, we found that overall perceived posttraumatic growth assessed in Wave 2 was robustly associated with improvements in one or more facet of each well-being domain (15/17 outcomes in total) assessed approximately six months later in Wave 3. Our findings suggest that perceived posttraumatic growth may contribute to individual well-being over the longer-term.

## Introduction

When people experience a potentially traumatic event, some develop trauma symptoms and others experience more short-term disruption in their lives but respond resiliently (Bonanno, 2005). Others experience posttraumatic growth (PTG; Tedeschi and Calhoun, 2004). PTG is positive change experienced as a result of the struggle with a major life crisis or traumatic event. PTG has been theorized to occur in five general areas (Tedeschi et al., 2017). First, some people who endure a life crisis feel that new opportunities have arisen from it. Second, relationships can be modified for the better and feelings of connection might be forged with others who have suffered similarly. Third, individuals can feel that their own strength has been proven or even increased. Fourth, some people come to believe that they can appreciate life more fully. Finally, some people feel a sense of deepening spirituality, even if their assumptive world might have been modified substantially.

Research has supported PTG in many areas, and that body of research has been summarized in numerous authored books (e.g., Calhoun and Tedeschi, 2013; Tedeschi et al., 2018) and edited volumes (e.g., Calhoun and Tedeschi, 2006). As research on PTG has gained worldwide visibility, it is possible that people experiencing traumas, or what Bonanno (2005) calls potentially traumatic events, might perceive that they have experienced PTG. Yet their perceptions might not be scientifically accurate, in that some people who make such attributions of PTG might not show *actual* positive changes in functioning (e.g., Frazier et al., 2009). For example, the attribution of having experienced PTG might reflect positive reinterpretation coping (or other types of secondary control) in response to experiencing a potentially traumatic event (e.g., benefit finding), even though actual post-event improvements in functioning have not transpired (Boals et al., 2022). These conceptual considerations highlight the importance of examining how perceived PTG relates to subsequent functioning.

Besides conceptual nuances, there are methodological challenges to establishing a clear understanding of the relationship between perceived PTG and actual functioning. As has been identified repeatedly in reviews and commentaries (e.g., Infurna and Jayawickreme, 2019; Jayawickreme et al., 2021), most of the existing research on perceived PTG is cross-sectional. Alongside other potential drawbacks to cross-sectional research on perceived PTG (e.g., self-reported change may not accurately reflect true change), with cross-sectional designs it is often impossible to rule out reverse causality. For example, in the case of a positive correlation between perceived PTG and satisfaction with life (e.g., Johnson and Boals, 2015), one is unable to determine whether perceived PTG leads people to experience greater life satisfaction or whether those who feel more satisfied with their lives tend to report greater perceived PTG.

Even when longitudinal studies have been conducted, perceived PTG is often examined in relation to a single or small set of outcomes, particularly psychological ones (Infurna and Jayawickreme, 2019). Assessing a broader range of outcomes could contribute to developing a more holistic and integrative understanding of how perceived PTG might be related to well-being. Moreover, much of the existing empirical literature on perceived PTG is from samples of people living in Western, Educated, Industrialized, Rich, and Democratic (WEIRD) societies, and concerns have been raised about the extent to which evidence from the current literature generalizes to people from other cultural contexts (Jayawickreme et al., 2021). Hence, further research is necessary to strengthen evidence about perceived PTG in cultures that are underrepresented in the psychological literature, particularly those in which historical climates of conflict could impact PTG processes and/or linkages between PTG and well-being (Bechara et al., 2021). Taken together, there is a need for additional longitudinal studies that establish a clear temporal order between perceived PTG and a wide range of well-being outcomes in less WEIRD contexts.

### The current study

In light of the abovementioned gaps in the existing literature, the present study uses three waves of data to examine associations of perceived PTG with a range of subsequent well-being outcomes among a sample of Colombian emerging adults experiencing distress following an interpersonal transgression in which they were hurt by another person. Our primary analysis involved estimating potential causal effects of overall perceived PTG (using the multidimensional Posttraumatic Growth Inventory-Short Form [PTGI-SF]; Cann et al., 2010) assessed in Wave 2 on 17 indicators of well-being across five domains of functioning (i.e., psychological well-being, psychological distress, social well-being, physical well-being, character strengths) assessed in Wave 3, adjusting for various potential confounders assessed in Wave 1. We expected that perceived PTG would generally be related to improved well-being approximately six months later, with some variation in the magnitude of associations across the outcomes. In a secondary analysis, we explored potential variation in associations across dimensions of perceived PTG by repeating the primary analysis for each of the five dimensions of perceived PTG assessed via the PTGI-SF.

## Materials and methods

### Study sample

Data for this study were taken from a three-wave longitudinal research project on interpersonal forgiveness and well-being among students (*N* = 2,878) attending a university located at Monteria, Colombia. Ethical approval was granted by Universidad del Sinú in Colombia. The Wave 1 assessment was completed from August 23 to September 9, 2021, with follow-up assessments completed approximately two months (Wave 2: October 25 to November 6, 2021) and six months later (Wave 3: February 7 to February 21, 2022). Except for sociodemographic and transgression-related characteristics that were only assessed in Wave 1, all variables were measured in each wave.

The analytic sample for this study comprised a subset of participants who met a multipronged set of criteria in Wave 1. We began by selecting participants for possible inclusion if they reported experiencing a potentially traumatic event in the form of an interpersonal transgression (Wade et al., 2014). Those who were transgressed against also had to meet cut-offs on two indices—self-rated transgression severity and subjective suffering—that we used to establish whether they were experiencing distress in the aftermath of the interpersonal transgression. Specifically, we included participants in the analytic sample who (1) reported that the transgression was at least slightly severe (a rating of ≥4/5 on a five-point response scale) and (2) endorsed at least a moderate degree of subjective suffering (a score of ≥4/10; Cowden et al., 2022b) on a six-item version of the Personal Suffering Assessment (VanderWeele, 2019). Together, these secondary criteria served as a proxy for experiencing interpersonal trauma. Of the *N* = 2,878 participants who completed the Wave 1 assessment, there were *n* = 636 participants who fulfilled all three criteria. These participants formed the analytic sample for this study.

The mean age of the analytic sample was 20.93 years (*SD* = 3.81), the majority of whom were female (72.80%), unmarried (82.39%), lived in a household in which at least one person earned a minimum wage (54.25%), and identified as religious (70.44%). Many types of interpersonal transgressions were reported (see Supplemental Table S1), the most common of which was verbal/emotional abuse (34.28%).

### Measures

#### Exposure

The exposure variable, perceived PTG, was taken from Wave 2. Participants completed the 10-item PTGI-SF (Cann et al., 2010). The items are evenly distributed across five subscales, including ‘relating to others,’ ‘new opportunities,’ ‘personal strength,’ ‘spiritual change,’ and ‘appreciation of life.’ In this study, we averaged responses to all items for an overall perceived PTG score. To obtain a more nuanced understanding of how the different dimensions of perceived PTG related to outcomes of interest, we also used the five subscales individually. The PTGI-SF items, response options, and estimated internal consistency of scores for overall perceived PTG and each dimension of perceived PTG can be found in Supplemental Table S2.

#### Outcomes

All outcome variables were assessed in Wave 3. We examined 17 outcomes across different domains of well-being, including psychological well-being (i.e., self-rated mental health, meaning in life, sense of purpose, happiness, life satisfaction), psychological distress (i.e., anxiety symptoms, depression symptoms, subjective suffering), social well-being (i.e., content with relationships, satisfying relationships, loneliness), physical well-being (i.e., self-rated physical health, sleep quality), and character strengths (i.e., state hope, trait forgivingness, orientation to promote good, delayed gratification). Details about the measures that were used to assess the outcomes, including the specific items, response options, and estimated internal consistency of scores (for multi-item measures), can be found in Supplemental Table S2.

#### Covariates

We controlled for several covariates assessed in Wave 1, including sex (female vs. male), household income (less than minimum wage vs. minimum wage or higher), marital status (unmarried vs. married), religious status (not religious vs. religious), frequency of religious service attendance (continuous), war survivor status (not a war survivor vs. war survivor), and financial/material stability (continuous) assessed using an average of two items from the Secure Flourishing Index (VanderWeele, 2017).

### Data analysis

All analyses were conducted using the *lavaan* package in R 4.1.3. A full-information maximum likelihood estimator was used to account for missing values. We followed the analytic template for outcome-wide longitudinal designs with observational data, which is a rigorous analytic approach for estimating potential causal effects of a single exposure on a wide range of subsequent outcomes (VanderWeele et al., 2020).

In our primary analysis, we performed a series of linear regressions in which continuous scores of each outcome assessed in Wave 3 were regressed on continuous scores of overall perceived PTG reported in Wave 2 (one outcome at a time). All models adjusted for each of the covariates, prior values of each outcome, and the prior value of overall perceived PTG assessed in Wave 1. By controlling for covariates assessed prior to the exposure variable, we avoid adjusting for covariates that may be on the pathway (i.e., mediators) from the exposure variable to one or more outcomes. Adjusting for prior values of the exposure and outcome variables can help to reduce concerns about reverse causation and further contribute to diminishing bias due to unmeasured confounding (VanderWeele et al., 2020). We standardized all outcomes (mean = 0, standard deviation = 1) to allow for effect sizes to be compared across the outcomes. As a secondary analysis, we repeated the primary analysis for each of the five dimensions of perceived PTG. Those models adjusted for the same set of covariates included in the primary analysis, prior values of all outcomes, and prior values of all five dimensions of perceived PTG assessed in Wave 1.

For both sets of analyses, we report the statistical significance (*p* < 0.05) of effect estimates both before and after applying Bonferroni corrections. Consistent with previous studies (e.g., Cowden et al., 2022a), we focus our interpretation on the unadjusted results because recommendations and practices of correcting for multiple testing vary and are constantly evolving. Based on the analytic approach used in the primary and secondary analyses, the results can be interpreted as the estimated effect of change in perceived PTG from Wave 1 to Wave 2 (or *incident* perceived PTG) on the change in each outcome from Wave 1 to Wave 3.

## Results

The results of the primary and secondary analyses are reported in Table 1. There was evidence of association between overall perceived PTG and almost all (15/17) of the outcomes. Robust associations were found with subsequent improvements in one or more outcomes on each of the well-being domains, including increases in the psychological well-being outcomes of self-rated mental health, meaning in life, sense of purpose, and life satisfaction (βs = 0.17 to 0.24, *p*s ≤ 0.001), a decrease in the psychological distress outcome of subjective suffering (β = −0.16, *p* = 0.002), increases in the social well-being outcomes of content with relationships and satisfying relationships (βs = 0.16 to 0.23, *p*s ≤ 0.002), and increases in all physical health outcomes (βs = 0.19 to 0.21, *p*s ≤ 0.001) and all character strengths outcomes (βs = 0.23 to 0.25, *p*s ≤ 0.001). Overall perceived PTG evidenced more modest associations with subsequent anxiety and depression symptoms (βs = −0.13 to −0.12, *p*s ≤ 0.022). Associations with outcomes of happiness (β = 0.09, *p* = 0.073) and loneliness (β = −0.08, *p* = 0.091) were more negligible.

**Table 1:** Associations of perceived posttraumatic growth (Wave 2) with subsequent outcome variables (Wave 3).

Outcomes	Overall perceived posttraumatic growth β [95% CI]	Perceived posttraumatic growth dimensions				
		Relating to others β [95% CI]	New opportunities β [95% CI]	Personal strength β [95% CI]	Spiritual change β [95% CI]	Appreciation of life β [95% CI]
*Psychological well-being*						
Mental health	0.19^**^ [0.10, 0.29]	0.16^**^ [0.07, 0.25]	0.16^**^ [0.07, 0.26]	0.19^**^ [0.10, 0.29]	0.16^**^ [0.06, 0.26]	0.11^*^ [0.02, 0.20]
Meaning in life	0.24^**^ [0.15, 0.34]	0.19^**^ [0.10, 0.28]	0.22^**^ [0.13, 0.31]	0.25^**^ [0.16, 0.34]	0.18^**^ [0.08, 0.28]	0.16^**^ [0.07, 0.25]
Sense of purpose	0.23^**^ [0.13, 0.32]	0.18^**^ [0.09, 0.27]	0.21^**^ [0.12, 0.30]	0.21^**^ [0.12, 0.30]	0.13^*^ [0.03, 0.23]	0.14^**^ [0.05, 0.23]
Happiness	0.09 [−0.01, 0.19]	0.11^*^ [0.01, 0.20]	0.07 [−0.03, 0.17]	0.09 [−0.01, 0.19]	0.02 [−0.09, 0.12]	0.09 [−0.01, 0.18]
Life satisfaction	0.17^**^ [0.07, 0.27]	0.14^**^ [0.05, 0.24]	0.16^**^ [0.06, 0.26]	0.18^**^ [0.08, 0.27]	0.09 [−0.02, 0.19]	0.13^*^ [0.04, 0.22]
*Psychological distress*						
Anxiety symptoms	−0.12^*^ [−0.22, −0.02]	−0.11^*^ [−0.20, −0.02]	−0.12^*^ [−0.22, −0.03]	−0.16^**^ [−0.25, −0.06]	−0.01 [−0.11, 0.09]	−0.11^*^ [−0.20, −0.02]
Depression symptoms	−0.13^*^ [−0.23, −0.03]	−0.12^*^ [−0.21, −0.02]	−0.15^**^ [−0.25, −0.05]	−0.16^**^ [−0.25, −0.06]	−0.03 [−0.13, 0.07]	−0.11^*^ [−0.20, −0.02]
Subjective suffering	−0.16^**^ [−0.26, −0.06]	−0.14^*^ [−0.23, −0.04]	−0.16^**^ [−0.26, −0.06]	−0.21^**^ [−0.30, −0.11]	−0.08 [−0.19, 0.02]	−0.08 [−0.18, 0.02]
*Social well-being*						
Content with relationships	0.23^**^ [0.13, 0.32]	0.18^**^ [0.09, 0.28]	0.22^**^ [0.13, 0.32]	0.28^**^ [0.19, 0.37]	0.11^*^ [0.01, 0.22]	0.13^*^ [0.03, 0.22]
Satisfying relationships	0.16^**^ [0.06, 0.25]	0.15^**^ [0.06, 0.25]	0.14^*^ [0.04, 0.24]	0.20^**^ [0.10, 0.29]	0.03 [−0.07, 0.14]	0.08 [−0.01, 0.18]
Loneliness	−0.08 [−0.18, 0.01]	−0.12^*^ [−0.22, −0.03]	−0.09 [−0.18, 0.01]	−0.12^*^ [−0.21, −0.02]	−0.05 [−0.15, 0.05]	−0.01 [−0.11, 0.08]
*Physical well-being*						
Physical health	0.21^**^ [0.12, 0.31]	0.18^**^ [0.09, 0.27]	0.17^**^ [0.07, 0.26]	0.22^**^ [0.13, 0.32]	0.20^**^ [0.10, 0.30]	0.11^*^ [0.02, 0.20]
Sleep quality	0.19^**^ [0.09, 0.29]	0.23^**^ [0.14, 0.32]	0.19^**^ [0.10, 0.29]	0.20^**^ [0.10, 0.30]	0.13^*^ [0.02, 0.23]	0.12^*^ [0.02, 0.21]
*Character strengths*						
State hope	0.25^**^ [0.15, 0.35]	0.19^**^ [0.10, 0.28]	0.21^**^ [0.12, 0.31]	0.26^**^ [0.17, 0.36]	0.13^*^ [0.03, 0.24]	0.16^**^ [0.07, 0.26]
Trait forgivingness	0.23^**^ [0.13, 0.32]	0.20^**^ [0.11, 0.29]	0.22^**^ [0.12, 0.31]	0.24^**^ [0.15, 0.33]	0.14^*^ [0.04, 0.24]	0.13^*^ [0.04, 0.22]
Orientation to promote good	0.23^**^ [0.14, 0.32]	0.19^**^ [0.10, 0.28]	0.23^**^ [0.14, 0.33]	0.23^**^ [0.14, 0.32]	0.12^*^ [0.02, 0.23]	0.15^**^ [0.05, 0.24]
Delayed gratification	0.24^**^ [0.14, 0.33]	0.21^**^ [0.12, 0.30]	0.22^**^ [0.12, 0.31]	0.26^**^ [0.16, 0.35]	0.12^*^ [0.01, 0.22]	0.15^**^ [0.05, 0.24]

*Note:* β, standardized estimate; CI, confidence interval. *n* = 636 for all analyses. In separate models, ordinary least squares regressions were used to regress each outcome assessed in Wave 3 on overall perceived posttraumatic growth (and each dimension of perceived posttraumatic growth) assessed in Wave 2. All models adjusted for sex, age, household income, marital status, religious status, frequency of religious service attendance, war survivor status, financial/material stability, and prior values of all outcomes assessed in Wave 1. Models with overall perceived posttraumatic growth as the exposure also adjusted for the prior value of overall perceived posttraumatic growth assessed in Wave 1. Models with one of the five perceived posttraumatic growth dimensions as the exposure also adjusted for prior values of all five dimensions of perceived posttraumatic growth assessed in Wave 1. *p < 0.05, **p < 0.003 (the *p*-value cut-off for Bonferroni correction of multiple tests for each outcome: 0.05/17 = 0.003).

In the secondary analysis, the pattern of associations for the dimensions of PTG were largely consistent with those that emerged for overall perceived PTG. Specifically, 10/15 outcomes that were predicted by overall perceived PTG also showed evidence of association with all five dimensions of perceived PTG. However, there were some differences compared to the general trend that was found for the associations involving overall perceived PTG. For example, overall perceived PTG was robustly associated with life satisfaction, subjective suffering, and satisfying relationships, but one or more dimensions of perceived PTG (e.g., ‘spiritual change’) evidenced a more negligible association with each of these outcomes. In contrast, happiness and loneliness were modestly associated with at least one dimension of perceived PTG (e.g., ‘relating to others’), even though overall perceived PTG evidenced a more negligible association with both of these outcomes. Only the ‘relating to others’ dimension was associated with all 17 outcomes, with slightly fewer associations found for ‘personal strength’ (16/17), ‘new opportunities’ (15/17), ‘appreciation of life’ (13/17), and ‘spiritual change’ (10/17).

## Discussion

In this study of young college-attending adults in Colombia, we used three waves of longitudinal data to examine associations of perceived PTG with 17 well-being outcomes assessed approximately six months later. Two key findings emerged. First, overall perceived PTG was associated with improvements in most well-being outcomes that we examined. There was some variability in the magnitude of effect sizes across the outcomes, with consistently stronger associations found across outcomes on some domains (i.e., character strengths) compared to others (i.e., psychological distress). Second, all dimensions of perceived PTG were associated with improvements in multiple well-being outcomes, although some dimensions (e.g., ‘relating to others’) were more consistently associated with the outcomes than others (e.g., ‘spiritual change’). Overall, our findings resonate with research that suggests positive changes in narrative identity (in this instance perceived PTG) have the potential to predict improvements in well-being (Adler et al., 2016).

Whereas much of the previous empirical literature on perceived PTG has focused on a single or narrow set of outcomes (Infurna and Jayawickreme, 2019), the current study provides insight into potential effects of perceived PTG on a wide range of outcomes across different domains of functioning. Although our findings suggest that perceived PTG is associated with improvements in many facets of well-being, effect sizes varied across the outcomes. For example, associations for overall perceived PTG ranged from very small (e.g., lower subsequent loneliness) to medium (e.g., higher subsequent state hope) in effect size (Funder and Ozer, 2019). Hence, perceived PTG may not have uniform implications for different facets of well-being, highlighting the importance of applying a more integrative and multidimensional approach to examining well-being in studies of perceived PTG.

Our secondary analysis indicated that the dimensions of perceived PTG varied in their associations with the well-being outcomes. For example, the dimension of ‘relating to others’ was associated with improvements in all subsequent outcomes. In contrast, ‘spiritual change’ showed little evidence of association with nearly half of the outcomes. Such findings align with previous research that has reported distinct impacts of different dimensions of perceived PTG (e.g., Frazier et al., 2009), indicating that some dimensions of perceived PTG may have more widespread implications for well-being than others. We also found that some outcomes were predicted by all five dimensions of perceived PTG (e.g., mental health, content with relationships, state hope), whereas others (including a number of outcomes for which overall perceived PTG showed evidence of association) were predicted by some dimensions of perceived PTG but not others (e.g., happiness, anxiety symptoms, loneliness). This pattern of findings suggests that some facets of well-being might be more ubiquitously impacted by multiple dimensions of perceived PTG compared to others. Potential variability in associations between different dimensions of perceived PTG and facets of well-being could be important for mental health professionals to consider as they provide support to clients they work with.

There are methodological limitations of this study. First, this study’s findings are based on university students in Colombia. Both limitations—students and Colombians—inhibit larger generalization. Second, the analytic sample included participants who reported being transgressed against by another person. Further study is needed to determine whether our findings replicate in Colombians (and other samples from less WEIRD societies) who have experienced other forms of potentially traumatic events (e.g., natural disasters, serious illness). Third, we relied exclusively on self-report measures, which may be subject to measurement error. Fourth, we applied a rigorous analytic approach that attempted to address concerns about confounding and reverse causation, but with observational data there is always a possibility that some combination of unmeasured confounding and statistical uncertainty might explain away the associations that were observed.

## Conclusion

In this relatively brief longitudinal study with Colombian emerging adults, we documented evidence indicating that perceived PTG is related to improvements in multiple facets of well-being. These findings provide some encouragement that perceived PTG could have longer-term benefits for individual well-being, and suggest that mental health professionals involved in treating clients who report a positive change in functioning in the aftermath of being transgressed against might be able to rely (to an extent) on their self-assessments during treatment. Although our findings extend the existing body of evidence on perceived PTG to a less WEIRD context that has experienced a long history of civil conflict, more research is needed to address the ongoing debate about the implications of perceived vs. actual PTG for individual well-being.

## Data availability statement

The data presented in this article will be made available after the embargo period. Requests to access the data should be directed to job.chen@uncc.edu.

## Ethics statement

Institutional approval to conduct this study was provided by the ethical review board at Universidad del Sinú in Colombia. The participants provided their written informed consent to participate in this study.

## Author contributions

ZC contributed to conceptualization, data analysis, and writing. AB contributed to conceptualization, data collection, and writing. RC contributed to conceptualization and writing. EW contributed to conceptualization and writing.

## Funding

This study was made possible through the support of a grant from Templeton World Charity Foundation, Inc. (grant number TWCF0390) and funding provided by Universidad del Sinú. The opinions expressed in this publication are those of the authors and do not necessarily reflect the views of Templeton World Charity Foundation, Inc.

## Conflict of interest

The authors declare that the research was conducted in the absence of any commercial or financial relationships that could be construed as a potential conflict of interest.

## Publisher’s note

All claims expressed in this article are solely those of the authors and do not necessarily represent those of their affiliated organizations, or those of the publisher, the editors and the reviewers. Any product that may be evaluated in this article, or claim that may be made by its manufacturer, is not guaranteed or endorsed by the publisher.
