# Supplementary material for: Perceived posttraumatic growth after interpersonal trauma and subsequent well-being among young Colombian adults: A longitudinal analysis
Source: Front Psychol. 2022 Nov 2;13:993609. doi: 10.3389/fpsyg.2022.993609 (PMC9667110; doi:10.3389/fpsyg.2022.993609)
Supplement: Supplementary file 1 [file Data_Sheet_1.pdf]

Perceived Posttraumatic Growth After Interpersonal Trauma and Subsequent Well-being Among Young Colombian Adults: A Longitudinal Analysis

SUPPLEMENTAL MATERIAL

Supplemental Table S1

Summary Statistics for Types of Transgressions

Transgressions	*n*	%
Infidelity	43	6.76%
Deception	57	8.96%
Broken promise(s)	21	3.30%
Disclosed a secret(s)	15	2.36%
Sexual abuse/violence	13	2.04%
Physical abuse/violence	28	4.40%
Verbal/emotional abuse	218	34.28%
Inappropriate communication/harassment	22	3.46%
Discrimination	30	4.72%
Act of war/violent hostility/rioting	7	1.10%
Gaslighting/distancing/ignoring	20	3.14%
Sabotage of social connections/defamation of reputation	7	1.10%
Threats to property/resources	4	0.63%
Accusations/moral affronts	11	1.73%
Indifference	25	3.93%
Harm to another person(s)	12	1.89%
Insensitive communication/behavior	14	2.20%
Other[a]	89	13.99%

Note. Cumulative percentage may exceed 100% due to rounding. [a]Includes transgressions that participants were unable to classify under any of the other specific transgression categories.

Supplemental Table S2

Summary of Measures

Subscale names	Scale items	Response scale	Variable type	Alpha
**Posttraumatic Growth Inventory-Short Form (Cann et al., 2010)**				
Overall perceived posttraumatic growth	All items listed below.	0 (*I did not experience this change as a result of my crisis*) to 5 (*I experienced this change to a very great degree as a result of my crisis*)	Continuous (average of the 10 items)	Wave 1: α = .91<br>Wave 2: α = .94
Relating to others	I have a greater sense of closeness with others.<br>I learned a great deal about how wonderful people are.		Continuous (average of the 2 items)	Wave 1: α = .75<br>Wave 2: α = .82
New opportunities	I am able to do better things with my life.<br>I established a new path for my life.		Continuous (average of the 2 items)	Wave 1: α = .80<br>Wave 2: α = .82
Personal strength	I know better that I can handle difficulties.<br>I discovered that I'm stronger than I thought I was.		Continuous (average of the 2 items)	Wave 1: α = .81<br>Wave 2: α = .85
Spiritual change	I have a better understanding of spiritual matters.<br>I have a stronger religious faith.		Continuous (average of the 2 items)	Wave 1: α = .82<br>Wave 2: α = .84
Appreciation of life	I changed my priorities about what is important in life.<br>I have a greater appreciation for the value of my own life.		Continuous (average of the 2 items)	Wave 1: α = .57<br>Wave 2: α = .68
**State Hope Scale (Snyder et al., 1996)**				
State hope	If I should find myself in a jam, I could think of many ways to get out of it.<br>At the present time, I am energetically pursuing my goals.<br>There are lots of ways around any problem that I am facing now.<br>Right now I see myself as being pretty successful.<br>I can think of many ways to reach my current goals.<br>At this time, I am meeting the goals that I have set for myself.	1 (*Definitely false*) to 8 (*Definitely true*)	Continuous (average of the 6 items)	Wave 1: α = .85<br>Wave 3: α = .92
**Trait Forgivingness Scale (Berry et al., 2005)**				
Trait forgivingness	People close to me probably think I hold a grudge too long.<br>I can forgive a friend for almost anything.<br>If someone treats me badly, I treat him or her the same.<br>I try to forgive others even when they don't feel guilty for what they did.<br>I can usually forgive and forget an insult.<br>I feel bitter about many of my relationships.<br>Even after I forgive someone, things often come back to me that I resent.	1 (*Strongly disagree*) to 5 (*Strongly agree*)	Continuous (average of the 10 items)	Wave 1: α =.78<br>Wave 3: α = .82

	There are some things for which I could never forgive even a loved one.<br>I have always forgiven those who have hurt me.<br>I am a forgiving person.			
**Brief Symptoms Inventory 18 (Derogatis, 2001)**				
Depression symptoms	Feeling no interest in things.<br>Feeling lonely.<br>Feeling blue.<br>Feelings of worthlessness.<br>Feeling hopeless about the future.<br>Thoughts of ending your life.	0 (*Not at all*) to 4 (*Extremely*)	Continuous (average of the 6 items)	Wave 1: α = .83<br>Wave 3: α = .92
Anxiety symptoms	Nervousness or shakiness inside.<br>Feeling tense or keyed up.<br>Suddenly scared for no reason.<br>Feeling so restless you couldn't sit still.<br>Spells of terror or panic.<br>Feeling fearful.		Continuous (average of the 6 items)	Wave 1: α = .87<br>Wave 3: α = .95
**Secure Flourishing Index (VanderWeele, 2017)**				
Life satisfaction	Overall, how satisfied are you with life as a whole these days?	0 (*Not satisfied at all*) to 10 (*Completely satisfied*)	Continuous (single item)	-
Happiness	In general, how happy or unhappy do you usually feel?	0 (*Extremely unhappy*) to 10 (*Extremely happy*)	Continuous (single item)	-
Physical health	In general, how would you rate your physical health?	0 (*Poor*) to 10 (*Excellent*)	Continuous (single item)	-
Mental health	How would you rate your overall mental health?	0 (*Poor*) to 10 (*Excellent*)	Continuous (single item)	-
Meaning	Overall, to what extent do you feel the things you do in your life are worthwhile?	0 (*Not at all worthwhile*) to 10 (*Completely worthwhile*)	Continuous (single item)	-
Purpose	I understand my purpose in life.	0 (*Strongly disagree*) to 10 (*Strongly agree*)	Continuous (single item)	-
Orientation to promote good	I always act to promote good in all circumstances, even in difficult and challenging situations.	0 (*Not true of me*) to 10 (*Completely true of me*)	Continuous (single item)	-
Delayed gratification	I am always able to give up some happiness now for greater happiness later.	0 (*Not true of me*) to 10 (*Completely true of me*)	Continuous (single item)	-
Content with relationships	I am content with my friendships and relationships.	0 (*Strongly disagree*) to 10 (*Strongly agree*)	Continuous (single item)	-

Satisfying relationships	My relationships are as satisfying as I would want them to be.	0 (*Strongly disagree*) to 10 (*Strongly agree*)	Continuous (single item)	-
Financial/material stability	How often do you worry about being able to meet normal monthly living expenses?<br>How often do you worry about safety, food, or housing?	0 (*Worry all of the time*) to 10 (*Do not ever worry*)	Continuous (average of the 2 items)	Wave 1: α = .88
	**UCLA Loneliness Scale (Russell et al., 1980)**			
Loneliness	How often do you feel that you lack companionship?<br>How often do you feel left out?<br>How often do you feel isolated from others?	1 (*Never*) to 3 (*Often*)	Continuous (average of the 3 items)	Wave 1: α = .81<br>Wave 3: α = .88
	**Single-item Sleep Quality Scale (Snyder et al., 2018)**			
Sleep quality	During the past 7 days, how would you rate the overall quality of your sleep?	1 (*Terrible*) to 5 (*Excellent*)	Continuous (single item)	-
	**Personal Suffering Assessment (VanderWeele, 2019)**			
Subjective suffering	The intensity of my suffering feels intolerable.<br>The length of time I have been suffering, or expect to suffer, feels intolerable.<br>I feel powerless to stop my current experience of suffering.<br>The suffering I have been experiencing affects all aspects of my life.<br>Certain purposes of my life have been badly disrupted because of the suffering I have been experiencing.<br>The suffering I have been experiencing threatens who I am as a person.	0 (*Strongly disagree*) to 10 (*Strongly agree*)	Continuous (average of the 6 items)	Wave 1: α = .81<br>Wave 3: α = .97

References

Cann, A., Calhoun, L. G., Tedeschi, R. G., Taku, K., Vishnevsky, T., Triplett, K. N., & Danhauer, S. C. (2010). A short form of the Posttraumatic Growth Inventory. *Anxiety, Stress, & Coping*, *23*(2), 127–137. https://doi.org/10.1080/10615800903094273

Russell, D., Peplau, L. A., & Cutrona, C. E. (1980). The revised UCLA Loneliness Scale: concurrent and discriminant validity evidence. *Journal of Personality and Social Psychology*, *39*(3), 472–480. https://doi.org/10.1037//0022-3514.39.3.472

Berry, J. W., Worthington, E. L., Jr., O'Connor, L. E., Parrott, L., III, & Wade, N. G. (2005). Forgivingness, vengeful rumination, and affective traits. *Journal of Personality*, *73*(1), 183–226. https://doi.org/10.1111/j.1467-6494.2004.00308.x

Derogatis, L. (2001). *Brief Symptoms Inventory 18: Administration, scoring, and procedures manual.* NCS Pearson.

Snyder, C. R., Sympson, S. C., Ybasco, F. C., Borders, T. F., Babyak, M. A., & Higgins, R. L. (1996). Development and validation of the State Hope Scale. *Journal of Personality and Social Psychology*, *70*(2), 321–335. https://doi.org/10.1037//0022-3514.70.2.321

Snyder, E., Cai, B., DeMuro, C., Morrison, M. F., & Ball, W. (2018). A new single-item sleep quality scale: Results of psychometric evaluation in patients with chronic primary insomnia and depression. *Journal of Clinical Sleep Medicine*, *14*(11), 1849–1857. https://doi.org/10.5664/jcsm.7478

VanderWeele, T. J. (2017). On the promotion of human flourishing. *Proceedings of the National Academy of Sciences of the United States of America, 114*(31), 8148–8156. https://doi.org/10.1073/pnas.1702996114

VanderWeele, T. J. (2019). Suffering and response: Directions in empirical research. *Social Science & Medicine, 224*, 58–66. https://doi.org/10.1016/j.socscimed.2019.01.041
